# Supplementary material for: Multi‐dataset identification of innovative feature genes and molecular mechanisms in keratoconus
Source: J Cell Mol Med. 2024 Sep 19;28(18):e70079. doi: 10.1111/jcmm.70079 (PMC11412914; doi:10.1111/jcmm.70079)
Supplement: Supplementary file 2 — Material S2. [file JCMM-28-e70079-s002.zip › 7.GSEA analysis/2.ATOH7/KEGG_GLYOXYLATE_AND_DICARBOXYLATE_METABOLISM.html]

Details for gene set KEGG\_GLYOXYLATE\_AND\_DICARBOXYLATE\_METABOLISM[GSEA]

|  || Dataset | ATOH7.group.cls#h\_versus\_l |
| Phenotype | group.cls#h\_versus\_l |
| Upregulated in class | l |
| GeneSet | KEGG\_GLYOXYLATE\_AND\_DICARBOXYLATE\_METABOLISM |
| Enrichment Score (ES) | -0.2976346 |
| Normalized Enrichment Score (NES) | -0.8573314 |
| Nominal p-value | 0.6119096 |
| FDR q-value | 1.0 |
| FWER p-Value | 1.0 |
Table: GSEA Results Summary

  

Fig 1: Enrichment plot: KEGG\_GLYOXYLATE\_AND\_DICARBOXYLATE\_METABOLISM      
 Profile of the Running ES Score & Positions of GeneSet Members on the Rank Ordered List

  

| SYMBOL | TITLE | RANK IN GENE LIST | RANK METRIC SCORE | RUNNING ES | CORE ENRICHMENT || 1 | HAO2 | na | 2717 | 0.298 | 0.1577 | Yes |
| 2 | ACO1 | na | 7702 | 0.125 | 0.1136 | No |
| 3 | MTHFD1L | na | 9237 | 0.095 | 0.1446 | No |
| 4 | MTHFD1 | na | 11350 | 0.061 | 0.1323 | No |
| 5 | MTHFD2L | na | 12922 | 0.041 | 0.1198 | No |
| 6 | ACO2 | na | 14683 | 0.024 | 0.0880 | No |
| 7 | MDH1 | na | 15209 | 0.019 | 0.0882 | No |
| 8 | HYI | na | 15291 | 0.019 | 0.1006 | No |
| 9 | MTHFD2 | na | 17477 | 0.000 | 0.0379 | No |
| 10 | CS | na | 17565 | -0.000 | 0.0356 | No |
| 11 | GRHPR | na | 18160 | -0.005 | 0.0227 | No |
| 12 | AFMID | na | 18650 | -0.010 | 0.0166 | No |
| 13 | MDH2 | na | 19066 | -0.013 | 0.0153 | No |
| 14 | PGP | na | 21425 | -0.036 | -0.0239 | No |
| 15 | GLYCTK | na | 30927 | -0.244 | -0.1039 | No |
| 16 | HAO1 | na | 31499 | -0.269 | 0.0930 | No |
Table: GSEA details [plain text format]

  

Fig 2: KEGG\_GLYOXYLATE\_AND\_DICARBOXYLATE\_METABOLISM      
 Blue-Pink O' Gram in the Space of the Analyzed GeneSet

  

Fig 3: KEGG\_GLYOXYLATE\_AND\_DICARBOXYLATE\_METABOLISM: Random ES distribution      
 Gene set null distribution of ES for **KEGG\_GLYOXYLATE\_AND\_DICARBOXYLATE\_METABOLISM**
